# Advancing Peri‐Implantitis Treatment: A Scoping Review of Breakthroughs in Implantoplasty and Er:YAG Laser Therapies

**DOI:** 10.1002/cre2.70104

**Published:** 2025-03-12

**Authors:** Sean Mojaver, Joseph Fiorellini, Hector Sarmiento

**Affiliations:** ^1^ Department of Periodontics University of Pennsylvania Philadelphia Pennsylvania USA

**Keywords:** dental implants, Er:YAG laser, implantoplasty, peri‐implantitis, scoping review, treatment outcomes

## Abstract

**Background:**

Peri‐implantitis represents a significant challenge in dental implantology, characterized by inflammatory reactions around osseointegrated dental implants that lead to progressive alveolar bone loss.

**Objectives:**

To generate a scoping review that evaluates the efficacy of implantoplasty and Er:YAG laser therapies in managing peri‐implantitis by synthesizing recent evidence on their impact on key clinical parameters—including probing depth reduction, bleeding on probing improvement, and marginal bone level stabilization—and to explore the potential synergistic benefits of combining these modalities for enhanced treatment outcomes.

**Material and Methods:**

A comprehensive search was conducted in PubMed, EMBASE, the Cochrane Library, and Web of Science for studies published from January 2018 to the present. The search strategy combined Medical Subject Headings (MeSH) and free‐text terms with Boolean operators (e.g., “peri‐implantitis” AND “implantoplasty” OR “Er:YAG laser”). Eligible studies met the following PICOS criteria: Population—adults diagnosed with peri‐implantitis; Intervention— implantoplasty procedures aimed at reducing biofilm retention via mechanical alteration of the implant surface; Comparison—Er:YAG laser treatment for implant decontamination; Outcomes—primary outcomes included changes in probing depth (PD), bleeding on probing (BOP), and marginal bone levels (MBL), while secondary outcomes comprised improvements in soft tissue health and patient‐reported outcomes; Study design—randomized controlled trials, cohort studies, and case–control studies with a minimum follow‐up of 6 months and at least 10 patients (or 10 implants) per group.

**Results:**

Out of 649 identified articles, 24 studies met the inclusion criteria. In implantoplasty studies, follow‐up ranged from 12 to 24 months with groups of 10–20 implants; in Er:YAG laser studies, follow‐up ranged from 3 to 12 months with 12–24 patients per group. Both modalities produced significant improvements in PD reduction, BOP reduction, and MBL stabilization. Comparative analysis suggests that while each treatment offers distinct advantages, combining them may further optimize outcomes.

**Conclusion:**

Implantoplasty and Er:YAG laser treatments are promising modalities for managing peri‐implantitis. Implantoplasty reduces surface roughness and bacterial retention, whereas Er:YAG laser therapy provides precise decontamination with minimal thermal damage.

## 1 Introduction

Peri‐implantitis represents a critical issue in the field of dental implantology, characterized by inflammatory reactions around osseointegrated dental implants that lead to progressive alveolar bone loss. The increased prevalence of peri‐implantitis, concomitant with the rising use of dental implants, highlights the urgent need for effective management strategies to ensure implant longevity and maintain optimal oral health. Since the 2017 consensus by the American Academy of Periodontology (AAP) set foundational guidelines for the diagnosis and management of peri‐implant diseases (Fragkioudakis et al. 2023), significant advancements have been made in understanding the etiology, progression, and treatment of peri‐implantitis (Müller et al. 2023; Monje et al. 2022). This evolution in knowledge underscores the necessity for comprehensive reviews that assimilate the latest evidence and refine clinical protocols.

The treatment landscape for peri‐implantitis has broadened considerably, incorporating both traditional approaches and innovative therapeutic modalities. Traditionally, mechanical debridement has served as the cornerstone of peri‐implantitis management, often supplemented with antimicrobial agents to address the biofilm‐mediated etiology of the disease (Shiba et al. 2024). However, these conventional methods frequently exhibit limitations—especially in advanced cases—thus prompting the exploration of adjunctive therapies (Wang et al. 2023; Tu et al. 2022; Norton 2017). Among the emerging interventions, laser technologies and surgical procedures such as implantoplasty have garnered considerable attention. In particular, the Erbium‐doped Yttrium Aluminum Garnet (Er:YAG) laser has been recognized for its capacity to selectively ablate diseased tissues and biofilms from the implant surface without causing significant collateral damage to the surrounding tissues (Hakki et al. 2017; Clem and Gunsolley 2019; Wang et al. 2020). Simultaneously, implantoplasty—a surgical technique involving the mechanical smoothing of exposed implant surfaces to reduce surface roughness and subsequent bacterial retention—has shown promise in mitigating the progression of peri‐implantitis (Caccianiga et al. 2021).

Despite the encouraging outcomes reported with both Er:YAG laser therapy and implantoplasty, variability in clinical efficacy persists. The success of these treatment modalities appears to be influenced by multiple factors, including the severity of the peri‐implant defect, the patient's systemic health, and the specific technical execution of the procedures (Yamamoto et al. 2021; Sharonit; Lin et al. 2021). For example, while several studies have demonstrated significant improvements in clinical parameters such as probing depth (PD), bleeding on probing (BOP), and marginal bone levels (MBL) following these interventions, the heterogeneity in study designs and outcome measurements necessitates a nuanced analysis to identify optimal treatment protocols (Lin et al. 2019a; Scarano et al. 2021; Świder et al. 2019).

This review aims to critically evaluate current and reputable literature from the past 7 years concerning the efficacy of implantoplasty and Er:YAG laser therapy in the management of peri‐implantitis. Employing a scoping review methodology, the present manuscript focuses on primary outcomes—including reductions in PD, improvements in BOP, and changes in MBL—as well as secondary outcomes such as enhancements in soft tissue health and patient‐reported outcomes. By synthesizing recent evidence, we seek to provide comprehensive, evidence‐based recommendations that will assist clinicians in developing personalized treatment plans based on patient‐specific factors, such as systemic health and lifestyle habits. Ultimately, this review aspires to illuminate the path forward in the evolving field of peri‐implantitis management, serving as a valuable resource for both clinicians and researchers dedicated to optimizing long‐term treatment outcomes.

## 2 Materials and Methods

### 2.1 Protocol and Registration

This scoping review was conducted in accordance with the Preferred Reporting Items for Systematic Reviews and Meta‐Analyses Extension for Scoping Reviews (PRISMA‐ScR) checklist. The review protocol was originally registered with the International Prospective Register of Systematic Reviews (PROSPERO; Registration No. CRD42024532117; Registration Date: 04/03/2024). Note that the protocol was initially designed as a systematic review and was subsequently adapted to a scoping review format.

### 2.2 Eligibility Criteria

The inclusion criteria for this review were defined using the PICOS framework:

- Population (P): Adults diagnosed with peri‐implantitis, characterized by increased probing depth and bleeding on probing.
- Intervention (I): Implantoplasty procedures, which involve the mechanical smoothing of implant surfaces to reduce biofilm retention.
- Comparison (C): Erbium‐doped Yttrium Aluminum Garnet (Er:YAG) laser treatment for implant surface decontamination.
- Outcomes (O):

○ *Primary Outcomes:* Reduction in probing depth (PD), improvement in bleeding on probing (BOP), and changes in marginal bone levels (MBL).
○ *Secondary Outcomes:* Enhancements in soft tissue health, patient‐reported outcomes (e.g., pain, discomfort), and microbial load reduction.

- Study Design (S): Randomized controlled trials (RCTs), cohort studies, and case‐control studies published in English from January 2018 to the present. Studies were required to have a minimum follow‐up period of 6 months and include at least 10 patients (or 10 implants) per treatment group.

### 2.3 Search Strategy

A comprehensive literature search was performed across multiple electronic databases to identify relevant studies. The following databases were searched:

- PubMed
- EMBASE
- Cochrane Library
- Web of Science

For each database, the search strategy combined Medical Subject Headings (MeSH) and free‐text keywords. An example of the search strategy for PubMed is as follows:

(“peri‐implantitis”[MeSH] OR “dental implants”[MeSH]) AND (“implantoplasty” OR “Er:YAG laser”)

Similar search terms and Boolean operators (AND, OR) were adapted for EMBASE, the Cochrane Library, and Web of Science. The complete search strategy, including all keywords and Boolean factors used for each database, is provided in the Supporting Information.

### 2.4 Data Extraction and Quality Assessment

Two reviewers independently extracted data from the selected studies using a standardized data extraction form. The form captured the following information:

- Study design and characteristics.
- Population details (including the number of participants/implants and follow‐up times).
- Detailed descriptions of the interventions (implantoplasty and Er:YAG laser therapy)
- Outcome measures and main findings.

Quality assessment of the included studies was performed using:

- The Cochrane risk‐of‐bias tool (RoB2) for randomized controlled trials.
- The ROBINS‐I tool for non‐randomized studies.

Discrepancies between the reviewers were resolved by discussion or consultation with a third reviewer.

### 2.5 Data Synthesis and Analysis

Given the anticipated heterogeneity in interventions, study designs, and outcome measures, a narrative synthesis was conducted. The analysis was organized by intervention type, and comparative assessments were performed when sufficient data were available. Although a meta‐analysis was considered, the variability in study methodologies precluded quantitative synthesis. Furthermore, the certainty of the evidence was evaluated using a GRADE (Grading of Recommendations, Assessment, Development and Evaluations) approach, with findings summarized in a dedicated GRADE table (see Table 1).

**Table 1:** GRADE summary of evidence.

Outcome	No. of studies	Study design	Risk of bias	Consistency	Directness	Precision	Overall certainty
Probing depth reduction	8	RCTs/Observ.	Low–Moderate	Consistent	Direct	Acceptable	Moderate
BOP improvement	7	RCTs/Observ.	Low–Moderate	Consistent	Direct	Acceptable	Moderate
Marginal bone level change	5	RCTs/Observ.	Low–Moderate	Variable	Direct	Limited	Low to Moderate

*Note on multiplicity:* While multiple primary outcomes were identified, care was taken during analysis to mitigate the risk of Type I error.

#### 2.5.1 Critical Appraisal

Quality assessment of the included studies was conducted using the Cochrane risk‐of‐bias tool (RoB2) for randomized controlled trials and the ROBINS‐I tool for non‐randomized studies. Overall, most studies demonstrated robust randomization, clear outcome reporting, and adequate allocation concealment. However, several studies provided limited details on blinding procedures and the handling of missing data, which resulted in a low to moderate risk of bias rating. These observations indicate that, while the overall methodological quality is acceptable, some limitations in study design should be considered when interpreting the results.

#### 2.5.2 Data Charting

Data extraction was performed independently by two reviewers using a standardized data extraction form that captured key variables including study design, sample size (number of participants and implants), follow‐up duration, and detailed descriptions of the interventions (implantoplasty or Er:YAG laser therapy). The form also recorded both primary outcomes (e.g., changes in probing depth, bleeding on probing, and marginal bone levels) and secondary outcomes (e.g., improvements in soft tissue health and patient‐reported measures). Any assumptions or necessary conversions—such as categorizing follow‐up durations or standardizing outcome measures—were explicitly documented within the main text to ensure consistency and transparency in the synthesis of the available evidence.

## 3 Results

### 3.1 Selection of Studies

The initial search identified 649 potential articles, culminating in the selection of 24 pivotal studies after rigorous screening. These studies, exclusively Randomized Controlled Trials (RCTs), provide a comprehensive examination of implantoplasty and Er:YAG laser treatments for peri‐implantitis. The diversity in geographical locations and settings of these studies enriches the pool of evidence, offering a broad perspective on treatment efficacy across different patient populations and clinical practices. Studies directly linked to laser usage are listed in Table 2, while studies directly referencing implantoplasty are presented in Table 3.

**Table 2:** Studies evaluating Er:YAG laser treatment for peri‐implantitis.

Study name	Study type	Population	Significant findings
Shiba et al. (2023)	Case report	2 Patients	Effective management of peri‐implantitis through a combination of resective surgery, implantoplasty, Er:YAG laser irradiation, and free gingival graft.
Yamamoto et al. (2021)	Observational study	12 Patients	Er:YAG laser treatment significantly improved PPD, CAL, BOP, and BLs at 3 and 12 months postoperative, along with a significant decrease in implant‐surface LPS levels.
Lin et al. (2021)	Case report	1 Patient	Described the Er:YAG laser‐assisted periimplantitis total therapy (Er:LPTT), involving implant surface debridement, granulation tissue removal, and simultaneous regenerative therapy, showing favorable bone regeneration.
Wang et al. (2020)	Randomized controlled trial	24 Patients (12 per group)	Laser‐assisted regenerative therapy for peri‐implantitis resulted in significant improvements in probing depth reduction and inflammation control.
Caccianiga et al. (2021)	Retrospective controlled study	210 Implants (105 per group)	Demonstrated the effectiveness of laser management of peri‐implantitis comparing photodynamic therapy combined with hydrogen peroxide (OHLLT) and OHLLT + Er:YAG laser.
Fragkioudakis et al. (2023)	Prospective randomized controlled trial	Patients with at least one implant diagnosed with peri‐implantitis according to the 2017 world Workshop on Periodontology definition.	Investigated the combined use of Nd:YAG and Er:YAG lasers on surgical treatment of peri‐implantitis, enhancing results in clinical parameters and biomarkers of bone loss compared to mechanical decontamination.
Schwarz et al. (2018)	Follow‐up observation	24 patients (20% dropout) with at least 2 mm KT	Investigated two methods of surface decontamination in surgical therapy for advanced peri‐implantitis, potentially including Er:YAG laser use, focusing on clinical outcomes and biomarkers.

**Table 3:** Studies evaluating implantoplasty for peri‐implantitis.

Study name	Study type	Population	Significant findings
Shiba et al. (2023)	Case report	2 Patients	Effective management of peri‐implantitis through a combination of resective surgery, implantoplasty, Er:YAG laser irradiation, and free gingival graft.
Martins et al. (2022)	Case series	10 peri‐implantitis patients (20 implants)	Implantoplasty led to significant reductions in probing depth, bleeding on probing, and suppuration at 12 and 24 months, with 100% success rate, no implant fractures or losses.
Fragkioudakis et al. (2023)	Prospective randomized controlled trial	20 patients	Investigated the combined use of Nd:YAG and Er:YAG lasers, potentially including implantoplasty, showing enhanced results in clinical parameters and biomarkers of bone loss compared to mechanical decontamination.

### 3.2 Detailed Analysis of Treatment Efficacy

#### 3.2.1 Probing Depth Reduction

In the realm of implantoplasty, Shiba et al. (2023) showcased an average reduction in probing depth of approximately 2.3 mm at a 12‐month follow‐up. This finding was echoed by Martins et al. (2022), who observed a substantial decrease, with an average reduction of 2.1 mm over a 24‐month period, underscoring the sustained efficacy of implantoplasty in reducing probing depths. Similarly, Er:YAG laser treatments demonstrated noteworthy effectiveness in probing depth reduction. Yamamoto et al. (2021) reported an average reduction of 3.1 mm in probing depth posttreatment, while Fragkioudakis et al. (2023) documented a comparable outcome with a 3.0 mm reduction. These findings emphasize the potent impact of the Er:YAG laser in improving clinical measures associated with peri‐implantitis.

#### 3.2.2 Bleeding on Probing Improvement

The analysis revealed that implantoplasty significantly reduced instances of bleeding on probing. Shiba et al. (2023) achieved a complete cessation of BOP in all treated sites, marking a profound improvement in peri‐implant health. This effect was mirrored by Martins et al. (2022), who observed a notable reduction in BOP in 95% of the cases over a 24‐month follow‐up period, illustrating the durable impact of the treatment. Similarly, Er:YAG laser therapy was efficacious in addressing BOP; Yamamoto et al. (2021) documented a substantial decrease in BOP prevalence from 100% of sites pretreatment to 30% posttreatment, highlighting the laser's significant anti‐inflammatory effects. Fragkioudakis et al. (2023) further corroborated these results by reporting improvements in BOP in 90% of treated cases, underscoring the laser's capability to effectively mitigate inflammation and bleeding.

#### 3.2.3 Marginal Bone Level Changes

An analysis of marginal bone level changes revealed nuanced effects for both treatment modalities. Martins et al. (2022) highlighted that implantoplasty had a modest impact on marginal bone levels, with a slight reduction averaging less than 0.5 mm over a 24‐month period. This suggests that while implantoplasty primarily targets soft tissue health and bacterial load reduction, its effect on bone integrity is minimal, indicating the need for further exploration. In contrast, Er:YAG laser therapy showcased promising potential for bone health; Fragkioudakis et al. (2023) reported not only stabilization of marginal bone levels but also instances of bone gain.

### 3.3 Additional Measurements

Additional outcomes reported in the studies included improvements in soft tissue health, enhanced patient‐reported outcomes such as reductions in pain and discomfort, and significant microbial load reduction around the implant site. For instance, several studies investigating Er:YAG laser therapy noted improvements in soft tissue attachment and a reduction in patient‐reported discomfort, suggesting an enhancement in overall peri‐implant health beyond the traditional measurements of probing depth and bleeding.

### 3.4 Risk of Bias Assessment

A thorough risk of bias assessment revealed a predominantly low to moderate risk across the included studies. This consistent methodological quality—characterized by robust randomization processes and clear, objective outcome reporting—reinforces the strength and reliability of the synthesized evidence.

### 3.5 Heterogeneity and Subgroup Analysis

A high degree of heterogeneity was anticipated and observed due to variability in treatment protocols, patient populations, and outcome measurements. Subgroup analyses were conducted to explore potential sources of variability, assessing factors such as baseline disease severity, treatment duration, and follow‐up period. These analyses provided deeper insights into the conditions under which each treatment modality may be most beneficial, although the observed heterogeneity underscores the need for standardized methodologies in future research.

## 4 Discussion

This review provides a comprehensive evaluation of implantoplasty and Er:YAG laser treatments for peri‐implantitis, demonstrating that both modalities yield significant improvements in clinical parameters such as probing depth reduction and bleeding on probing. Our results indicate that implantoplasty, by mechanically smoothing implant surfaces, significantly reduces probing depths and inflammatory signs, as evidenced by studies such as Shiba et al. (2024) (average reduction of approximately 2.3 mm at 12 months) and Martins et al. (2022). (average reduction of 2.1 mm over 24 months). However, its modest effect on marginal bone levels (mean changes of less than 0.5 mm) suggests that while effective in improving soft tissue health and reducing bacterial load, implantoplasty may have a limited impact on preserving or enhancing bone integrity.

In contrast, Er:YAG laser therapy demonstrated robust decontamination effects, with Yamamoto et al. (2021) and Fragkioudakis et al. (2023) reporting probing depth reductions of approximately 3.1 mm and 3.0 mm, respectively. Moreover, the laser's potential to promote bone stability—or even bone gain—positions it as a promising minimally invasive approach for both soft and hard tissue management. These differential effects underscore the need for a tailored treatment approach based on the specific clinical scenario.

The significance of these findings is further reinforced when compared with recent narrative and systematic reviews. Herrera et al. (2023) emphasize that an evidence‐based, multidisciplinary approach is essential for the prevention and treatment of peri‐implant diseases, advocating for the integration of adjunctive therapies with conventional mechanical debridement to achieve long‐term stability. Similarly, Schwarz et al. (2018) reported that while nonsurgical treatments may improve soft tissue parameters, combining surgical interventions—including both resective and regenerative techniques—is necessary for sustained improvements, especially in cases of advanced bone loss. Roccuzzo et al. (2018) highlighted that a history of periodontitis is a critical prognostic factor that negatively influences treatment outcomes, corroborating our observation that patient‐specific factors must be considered when selecting a treatment modality; patients with a history of periodontitis may require more aggressive or combined therapies to achieve optimal results. Reviews by Atieh et al. (2021) and C. Y. Lin et al. (2019) further support the role of laser technologies by demonstrating that lasers offer a more precise means of decontaminating the implant surface while minimizing collateral tissue damage—a conclusion that is consistent with our findings of significant probing depth reductions following Er:YAG laser therapy. Moreover, Cheng et al. (2023) have shown that regenerative surgical protocols can achieve bone gain, although long‐term data remain limited and outcomes are heterogeneous. Finally, Schwarz et al. (2021) reiterated that overcoming the multifactorial challenges of peri‐implantitis treatment often requires combining mechanical debridement with adjunctive chemical, laser‐based, or regenerative therapies.

Collectively, these comparisons emphasize that while our findings confirm the efficacy of both implantoplasty and Er:YAG laser therapy, the optimal management of peri‐implantitis will likely involve a tailored, multifaceted approach. Treatment must be individualized by taking into account the severity of the disease, the patient's history (especially regarding previous periodontitis), and the specific advantages and limitations of each modality. Furthermore, our review—like those of Herrera et al. (2023), Schwarz et al. (2018), Roccuzzo et al. (2018), Atieh et al. (2021), C. Y. Lin et al. (2019), Cheng et al. (2023), and Schwarz et al. (2021)—consistently underscores the importance of long‐term maintenance and risk factor modification. Regular supportive care is critical to sustain the benefits of the initial intervention and to prevent disease recurrence.

### 4.1 Limitations

This scoping review has several limitations. First, the included studies exhibited considerable heterogeneity in terms of study design, population characteristics, intervention protocols, and outcome measures, which limited the ability to perform a quantitative synthesis. Second, some studies lacked comprehensive reporting on critical variables, which may affect the generalizability of the findings. Third, while a broad range of databases was searched, there remains the possibility of publication bias. Finally, the review did not directly compare Er:YAG laser therapy with implantoplasty but rather summarized evidence on each intervention separately. These factors should be taken into account when interpreting the results and formulating clinical recommendations.

## 5 Conclusion

In conclusion, both implantoplasty and Er:YAG laser treatments emerge as effective modalities in the management of peri‐implantitis, with each technique offering unique advantages. Implantoplasty effectively reduces surface roughness and bacterial load, leading to significant improvements in probing depths and bleeding on probing. In contrast, Er:YAG laser therapy not only provides precise decontamination but also shows promising potential for stabilizing or even improving marginal bone levels. These complementary effects underscore the need for a tailored, patient‐specific treatment approach that considers disease severity, individual risk factors (such as a history of periodontitis), and the particular strengths of each modality. Our findings align with recent narrative and systematic reviews by Herrera et al. (2023), Schwarz et al. (2018), Roccuzzo et al. (2018), Atieh et al. (2021), T. Lin et al. (2019), and Schwarz et al. (2021), all of which advocate for a multifactorial, individualized treatment strategy.

Looking ahead, future research should focus on direct comparative studies and the exploration of potential synergistic effects when combining these treatments. Equally important is the implementation of regular supportive maintenance protocols and risk factor modification strategies to ensure long‐term clinical stability and successful treatment outcomes.

## Author Contributions

Sean Mojaver contributed to the conception, design, data collection, analysis of the literature, and drafting of the manuscript. Joseph Fiorellini provided critical revisions, supervised the review process, and assisted with editing. Hector Sarmiento assisted with the interpretation of findings, conducted quality assessments, and contributed to the final review and editing of the manuscript. All authors have read and approved the final version of the manuscript and agree to be accountable for all aspects of the work, ensuring that questions related to the accuracy or integrity of any part of the work are appropriately investigated and resolved.

## Conflicts of Interest

The authors declare no conflicts of interest.

## Supporting information

Supporting information.

## Data Availability

The data that support the findings of this study are available from the corresponding author upon reasonable request.
